# An evaluation framework for comparing geocoding systems

**DOI:** 10.1186/1476-072X-12-50

**Published:** 2013-11-08

**Authors:** Daniel W Goldberg, Morven Ballard, James H Boyd, Narelle Mullan, Carol Garfield, Diana Rosman, Anna M Ferrante, James B Semmens

**Affiliations:** 1 Department of Geography, Texas A&M University, College Station, Texas, USA; 2 Centre for Population Health Research, Curtin University, Perth, Western Australia, Australia; 3 Cooperative Research Centre for Spatial Information, Perth, Western Australia, Australia; 4 Data Linkage Branch, Western Australia Department of Health, Perth, Western Australia, Australia

**Keywords:** Geocoding, Georeferencing, Record linkage, Postal address data, Health records

## Abstract

**Background:**

Geocoding, the process of converting textual information describing a location into one or more digital geographic representations, is a routine task performed at large organizations and government agencies across the globe. In a health context, this task is often a fundamental first step performed prior to all operations that take place in a spatially-based health study. As such, the quality of the geocoding system used within these agencies is of paramount concern to the agency (the producer) and researchers or policy-makers who wish to use these data (consumers). However, geocoding systems are continually evolving with new products coming on the market continuously. Agencies must develop and use criteria across a number axes when faced with decisions about building, buying, or maintaining any particular geocoding systems. To date, published criteria have focused on one or more aspects of geocode quality without taking a holistic view of a geocoding system’s role within a large organization. The primary purpose of this study is to develop and test an evaluation framework to assist a large organization in determining which geocoding systems will meet its operational needs.

**Methods:**

A geocoding platform evaluation framework is derived through an examination of prior literature on geocoding accuracy. The framework developed extends commonly used geocoding metrics to take into account the specific concerns of large organizations for which geocoding is a fundamental operational capability tightly-knit into its core mission of processing health data records. A case study is performed to evaluate the strengths and weaknesses of five geocoding platforms currently available in the Australian geospatial marketplace.

**Results:**

The evaluation framework developed in this research is proven successful in differentiating between key capabilities of geocoding systems that are important in the context of a large organization with significant investments in geocoding resources. Results from the proposed methodology highlight important differences across all axes of geocoding system comparisons including spatial data output accuracy, reference data coverage, system flexibility, the potential for tight integration, and the need for specialized staff and/or development time and funding. Such results can empower decisions-makers within large organizations as they make decisions and investments in geocoding systems.

## Introduction

Across the world, individuals, research groups, and organizations of all sizes ranging from non-profit and commercial entities to local-, state- and national-level government agencies are often required to perform geocoding for numerous mission critical tasks [1,2]. Within a health context, geocoding – the process of converting textual information describing a location into one or more digital geographic representations – is used for such diverse processes as linking the individual-level addresses associated with health records to census enumeration units for disease surveillance at state and national levels, to determining individual levels exposures to environmental contaminants and identifying the accessibility of healthy food choices for populations of interest [3-10].

The person or organizational group responsible for performing the geocoding is faced with a number of choices regarding which geocoding system to employ to achieve a result that is sufficient for the purposes intended [11-15]. These choices may greatly impact the results of the geocoding process in terms of output data quality which propagates to subsequent studies that utilize these geocoded data as input [4,7,11,12,14,16-22]. Despite the best intentions of those responsible for providing geocoded data, many of the choices may be conditioned by the constraints of the organization for whom or within which the geocoded data are produced. Within large organizations such as state and national Health Departments and Disease Registries, for example, existing operational workflows, data confidentiality requirements, and strategic partnerships between external organizations and agencies may preclude a geocoding system from being purchased, implemented, or integrated [4,6,23-25].

As can be expected with any mission-critical operational task, evaluating the factors that one might use to determine which geocoding system will best meet the needs of a specific organization or individual is often not a simple matter. Likewise, these factors may not be readily transferrable from one situation to another [2]. Switching between geocoding systems represents the potential for expending significant levels of time, effort and funding due to the need to integrate a new system within existing production workflows, perform evaluation testing, re-train staff, etc. Given these up-front and continuing costs, the decision to change geocoding systems is generally not entered into lightly. An evaluation which compares the benefits versus the costs of each geocoding system is a useful way of determining which geocoding system is the correct choice for a particular individual, group, or organization given the specific scenario within which it must operate and the user-base it should serve.

In recent years, several research groups have undertaken studies which have evaluated and compared the performance of various geocoding systems. Contributions to the body of geocoding literature have included evaluations of the spatial accuracy and match rates of geocoded systems resulting from the use commercially available systems versus in-house custom-built solutions [4,16,21,26,27]; the use of various forms of reference data files - building centroids, address points, areal units describing parcels, street centerline files, etc. [7,13,18,22,28-31]; and the use of different interpolation algorithms - address range, uniform lot, population weighted centroids, geographic imputation, etc. [18,22,32,33]; the use of different feature matching methods - probabilistic, deterministic, etc. [28,34-37]; the use of pre-processing techniques - address standardization, normalization, etc. [38]; and the use and effectiveness of manual/clerical review processes to improve non-matchable addresses [39].

Other authors have investigated the non-random effects that an urban, rural, or remote geographic context plays on the accuracy, completeness and correctness of input address data, reference data layers and ultimate geocode output [5,16,26,40-43]. Similarly, research has investigated the non-random distribution of geocoding quality by demographic characteristics such as race, ethnicity, and income [11,43-45].

This rich body of prior work into geocoding comparisons has provided valuable insight into the role that various components of a geocoding system play in the quality of output produced and the effect these choices may have on subsequent research projects [3,46]. However, despite this diverse set of resources that detail the factors which influence geocode quality, there remains a lack of up-to-date guidance that an organization or individual could use to assist in the determination of which geocoding system is right for a particular application/usage context. In particular, these prior studies have not considered the particular operational, technical, policy and legal issues that are present in large organizations responsible for securely collecting, linking, curating, producing and/or disseminating health-related geocoded data such as state-level Health Departments and Disease Registries [6,23,34]. Given that any number of high-quality commercial off the shelf (COTS) geocoding systems are now available, this issue is particularly relevant if the data maintained by these agencies are to be employed to the their full potential to best serve the public at large.

The primary purpose of this study is to develop an evaluation framework to assist a large organization to determine which geocoding systems will meet its operational needs. The decision criteria presented offer an enumeration of the capabilities that a government agency can consider, ranging from the most basic principles of how the software gets installed to advanced requirements such as the flexibility of the system for allowing specialized matching rules for particular types of input data. The framework developed is applicable to organizations of all sizes across all regions of the world. The example used herein provides the upper bound of requirements in terms of operational needs and confidentiality requirements. While it is expected that, globally, similar agencies would have comparably high-level needs, individual researchers working on small-scope projects may not require such stringent requirements and as such would be able to make different choices than those required at an agency responsible for safeguarding confidential information.

However, to be clear, the current research does not recommend the usage of any particular geocoding system; instead, it offers a methodology and a set of criteria by which an organization or individual could make such a decision for themselves. The strengths and weaknesses of the proposed approach are evaluated through this case study in Western Australia. In this case study, none of the geocoding systems evaluated is listed by name due to non-disclosure agreements with the vendors who participated in the study.

The remainder of this paper is organized as follows. We first develop the evaluation framework by defining several axes of criteria by which a geocoding system can be characterized and measured. Within each, specific examples of capabilities, constraints, and features are provided. We next describe the context within which the current evaluation was performed. This includes the general characteristics of the types of input data, geocoding systems, and reference data that were used. Only general characteristics are provided because of the confidential nature of the data processed and non-disclosure agreements. This limits the specific details that can be reported about the geocoding systems evaluated and the data tested, but nonetheless provides an opportunity to evaluate the proposed approach. Following the descriptions of the data and systems used, we present the results of the evaluation process and offer a discussion as to their meaning.

### Evaluation framework

The evaluation framework developed and used to facilitate the experiments contained herein is a combination of traditional geocoding system performance tests (match rates, spatial variation, etc.) and a series of evaluations which capture the applicability of a geocoding system to a particular user scenario (workflow integration, cost, etc.). While both aspects are important, the combination of the two serves to highlight the balance that must be struck between performance and utility in order for an organization to decide upon an appropriate system given the requirements, limitations and constraints of any particular organization or individual.

### Geocode quality

Output geocode quality is a primary concern for geocoded data producers and end-users of these data. Table 1 lists the typical metrics used to measure geocode quality. These are: (a) ***match rates*** – the proportion of input data a geocoding system was capable of successfully geocoding; (b) ***match type*** – the level of geographic match for a geocode (parcel, street centroid, postcode, etc.); (c) ***match score*** – the level of similarity between the input address data requested and the reference geographic feature matched to; and (d) ***spatial accuracy*** – the distance between the true location and the computed geocode location [2,6,34,47,48]. In addition to these, ***administrative unit concordance*** is often used to indicate cases where two geocoding systems (or different configurations of the same system) result in the assignment of differing administrative unit codes.

**Table 1:** Geocoding system quality metrics

**Quality Metric**	**Description**
**Match rate (%)**	Percentage of all records capable of being geocoded
**Match type (% by geographic level)**	Geographic levels of geocode match – building level, parcel level, street centroid level, postcode level, etc. and percentages of matchable geocodes at each level
**Match score (% at score levels)**	Frequency distribution of match scores for matchable geocodes
**Spatial accuracy (% at distance levels)**	Frequency distribution of distances between matchable geocodes and ground truth locations
**Spatial accuracy variation (% variation from other systems)**	Frequency distribution of distances between the same geocode produced by multiple geocoding systems.
**Administrative unit concordance (% variation from other systems)**	Frequency distribution of administrative unit concordance between the same geocode produced by multiple geocoding systems.

In the current study, the first three of these metrics were measured directly for each of the geocoding system configurations (i.e., combinations of input data, geocoding system, and reference data). Ground truth GPS points were not available for this research, so variation metrics were computed and reported for spatial accuracy and administrative unit concordance. Instances of high variation between geocoding configurations for particular addresses were used to guide the investigation of individual addresses that performed differently between geocoding configurations. Census unit concordance was not evaluated.

### Geocoding system operating characteristics

The integration of a new geocoding system within an organization potentially represents a great deal of time, effort, and training, among other costs. Thus the decision to scrap an old system and integrate a new one is generally not made lightly. As noted above, the qualities of geocode output (match rate, spatial accuracy, etc.) are but one of the axes by which a geocoding system must be evaluated when considering the adoption of a geocoding system at an organizational level. The applicability of a geocoding system to a particular user scenario (workflow integration, cost, etc.) is paramount in the decision to adopt a new system. A brief overview of the categories and a few example metrics related to geocoding system operation that can be used to compare the applicability and appropriateness of geocoding systems to a particular set of user needs and usage scenarios are displayed in Table 2; each is discussed in detail in the following sections. These listing are not intended to be exhaustive because different organizations will have different needs.

**Table 2:** Geocoding system operational capabilities metrics

**Category and notes**	**Metric**	**Description and/or example**
**System Flexibility**	User defined reference layers	Is it possible to use any available reference data
***Ability of the user of the system to make changes and additions to data sources and methods used by the system***	Specialized address parsing	Add in support for new street types, named places
	Specialized matching algorithms	Consider neighboring areas for matches
	Customized feature hierarchies	Hierarchy based on organizational policy
**System Integration**	Operating system support	Windows, Unix/Linux/Solaris
***Ability to merge a geocoding system into an existing production system***	System/Workflow Integration	Into tools and systems used by the organization (eg SAS wrappers)
	Varying operational modes	Batch/interactive/manual
	Desktop Version	Standalone product for highly sensitive data
	In-house Server Version	Internal server for multiple users within agency firewall
	API Version	Vendor or custom-written code for off-site processing
**Metadata**	Spatial confidence values	Descriptions of the region size (geographic area) that a geocode output is known to fall within
***Types and level of detail reported about the quality of the output data and/or the characteristics of the geocoding system***	Input address/matched address concordance	Descriptions of which attributes of the input address were incorrect, incomplete, partially matched or not used in the matching process
**Capabilities**	Automatic batch geocoding	The ability to process a data file of records using a single process
***Baseline functionality of paramount importance to agencies working with large health data sets***	Interactive review	The ability to perform manual review for non-matched records to attempt to determine a correct output geocode
	Alias tables	The ability to incorporate tables of named places, common synonyms for street address attributes
	Weighted centroids	The ability to bias the output location of a geocode based a known distribution of a characteristic of interest such as the distribution of population or specific subsets of a population in an area

### System flexibility

The flexibility of a geocoding system describes the ability of the user of the system to make changes and additions to the data sources and methods used by the system (Table 3). In this evaluation, flexibility was determined by the ability of a geocoding system to: (a) permit the utilization of ***user****-****defined reference data layers*** (points, lines, and polygons), e.g., import and use any reference data sources; (b) create and use ***specialized address parsing rules***, e.g., add in support for new street types, named places, etc.; (c) create and use ***specialized matching algorithms***, e.g., look in neighboring postcodes or localities for matches; and (d) the ability to create and use ***specialized feature selection hierarchies*** based on organizational policies, e.g., search in parcels first, then localities or postcodes, or alternatively, choose whichever has the smaller area.

**Table 3:** Geocoding system flexibility metrics

**Flexibility Metric**	**Description**
**User-defined reference data layers (Y/N)**	Does the user have the ability to include his/her own custom reference data layers? Example – including one’s own parcel layer for a locality if it is available.
**Specialized address parsing rules (Y/N)**	Does the user have the ability to include his/her own custom address parsing rules? Example – including a parsing approach where the “St.” in “St. Patrick” is converted to “Saint” to provide higher match rates given a reference data source that has the term listed as “Saint”.
**Specialized matching algorithms (Y/N)**	Does the user have the ability to include his/her own custom matching rules? Example – Inspecting nearby postal codes for similarly named streets and providing a higher matching score for candidate match features that are found in adjacent postal codes and lower match scored for candidate match features found in non-adjacent postal codes.
**Specialized feature selection hierarchies (Y/N)**	Does the user have the ability to include his/her own custom ordering of reference layers? Example – Adding the ability to search first in postal codes then in municipalities in urban regions (where postal codes are small and municipalities are big) and municipalities first then postal codes in rural regions (where municipalities are small and postal codes are large).

### System integration

The ability to merge a geocoding system into an existing production system is a major concern for large organizations that routinely perform geocoding as one aspect of a larger data processing system. Examples include the Western Australia (WA) Department of Health (DoH), Data Linkage Branch where the current study was undertaken. This group is responsible for providing data linkage services that consolidate data from numerous health-related sources for data consumers within the WA DoH and other local-, state-, and national-level agencies to facilitate research and policy-making. Geocoding services are provided as data are processed (linked) in order to associate census enumeration unit values with each record as part of the larger data linkage process. Similar systems are found in other Health Departments at the local-, regional-, state-, and national levels around the world, as well as Disease Registries where data consolidation, cleaning, and/or linkage tasks take place. In each scenario, the geocoding component of the overall organizational mission is tightly integrated into other dependent workflows. The geocoding process occurs as data are streamed through the system or in a batch-mode fashion from which the results are linked back to the output linked/consolidated records.

Table 4 lists the primary concerns for these organizations in terms of system integration. These are: (a) ***operating system support*** – the geocoding system must be executable on the operating systems used by the organization (Windows, Unix/Linux/Solaris, etc.); (b) ***system and workflow integration*** – the geocoding system should be integrateable with the tools and systems used by the organization (SAS wrappers, COM components, dynamically linked libraries, APIs, etc.); (c) ***operational modes*** – the geocoding system must be usable in the modes necessary to support the organizational mission (batch-mode, interactive-mode, manual review/rematching, etc.).

**Table 4:** Geocoding system integration metrics

**Integration metric**	**Description**
**Operating system support (Y/N)**	Does the system work on the operating system used by the organization? Example – Windows, Linux, Unix.
**System and workflow integration (Y/N)**	Can the system be integrated into existing systems and workflows used by the organization? Example – A system that can be wrapped as a SAS component so it can be integrated into automated SAS data processing workflows already used by the organization.
**Operational mode integration – Batch mode (Y/N)**	Does the system have the ability to geocode records in batch? Example – Uploading a large data set to a server and running the geocoding process over the whole file.
**Operational mode integration – Interactive mode (Y/N)**	Does the system have the ability to allow a user to interactively geocode records? Example – Displaying an interface that allows a user to geocode one record at a time.
**Operational mode integration – Manual review mode (Y/N)**	Does the system have the ability to allow a user to interactively geocode records that do not process correctly in batch mode? Example – Displaying an interface that lists records that did not match in batch processing and allows the user to research, correct, and re-geocode individual records one-by-one.

Table 5 lists various system interface modes that a geocoding platform could provide to a user. These refer to the ways in which a user would interact with the system. These interface modes are important because ***data security and/or confidentiality constraints*** may dictate certain forms of data processing. For example, it is the case that most health-related records cannot be transmitted outside of the secure environment within which they are housed so ***desktop or in-house server geocoding*** platforms may be the only option. In contrast, it may be acceptable to transmit non-confidential data over the Internet for offsite processing on a vendor’s servers through an ***application programmer interface (API)*** using custom-written code or a vendor-provided thin client.

**Table 5:** Geocoding system interface metrics

**Interface metric**	**Description**
**Desktop-based geocoding (Y/N)**	Does the system work on a desktop computer?
**Server-based geocoding (Y/N)**	Does the system work on a server?
**Application programmer interface (API) geocoding (Y/N)**	Does the system provide an API for which custom programs can be developed?

Such APIs and other online batch-process geocoding services where users can upload a database of addresses and have them geocoded by web-based services can be categorized as web-based geocoding options. Many commercial providers offer these services such as the APIs available from Google, Yahoo and Esri [49-51]. There are other similar community-specific geocoding services like those offered by the North American Association of Central Cancer Registries (NAACCR) created to meet the needs of specific research and practice communities [52]. Within the context of health data specifically, organizations must be able to ensure data privacy, security, and confidentiality through data confidentiality and use agreements with these service providers. Current research into cyber-enabled GIS infrastructures (CyberGIS) [53] as well as secure computing environments for health data [54] is broadening the scope of what is considered acceptable. However, it is the case that in some instances, health organizations may be specifically prohibited from using web-based geocoding services. At the time this study was performed, the organization performing this study had this restriction in place. Therefore web-based geocoding systems were not included in the present evaluation.

### Cost

The true cost of a geocoding system can be a difficult thing to quantify. However, some aspects of the geocoding system cost are easy to quantify. The price for a ***software license for the geocoding system***, the price of a ***license for the required reference data layers***, and the ***price for a support contract*** are examples of one-time (or yearly) fixed costs that can readily be obtained from a software vendor or assumed to be zero for open source software (Table 6). Each of these costs is a common expense in jurisdictions around the world, although there are free geocoding systems such as geocoder.us ^a^ , free reference data layers such as the US Census Bureau TIGER/Line files ^b^ , and unsupported geocoding systems such as the Postal Address Geocoder (PACG) ^c^ . However, others components that must be considered when estimating overall cost are more complicated because they involve computing time and effort for staff members.

**Table 6:** Geocoding system cost metrics

**Cost metric**	**Description**
**Software license cost ($)**	Price of licensing the software
**Reference data layer cost ($)**	Additional costs for licensing reference data
**Support contract cost ($)**	Cost of support contract
**FTE support cost ($)**	Cost of full time equivalent support (in-house)
**FTE development cost ($)**	Cost of full time equivalent development (in-house)
**FTE maintenance cost ($)**	Cost of full time equivalent maintenance (in-house)
**FTE training cost ($)**	Cost of full time equivalent training (in-house)
**FTE specialized skills (Y/N)**	Full time equivalent specialization required (in-house)

Table 6 lists these costs, which include: (a) the level of effort and/or number of full time equivalent positions (FTE) required to ***support the geocoding system*** – e.g., time/effort for a staff member to identify, respond to, and/or fix errors reported by end-users; (b) the level of effort and/or number of FTE required to ***develop the geocoding system*** – e.g., time/effort for a staff member to build additional components into the geocoding system as needed; and (c) the level of effort and/or number of FTE required to ***maintain the geocoding system*** – e.g., time/effort for a staff member to update the system to use the latest reference data files.

When purchasing a commercial, off-the-shelf (COTS) package, many of these items disappear because the ***vendor may charge fees*** to provide them to the customer; however, the flexibility of the geocoding system may decline because the vendor may not be capable of building in all of the custom functionality required by the user. In contrast, when building and using a custom in-house geocoding solution, flexibility is maximized, but it requires the ***availability and retention of specialized staff*** with particular training and familiarity with the geocoding system and the programming languages and programming environments upon which it is built. In evaluation performed in this report, cost is not considered as a factor due to non-disclosure agreements with the vendors who participated. However, when the framework described herein is used to make geocoding decisions within an organization, it is expected that cost would be a highly weighted metric.

### Metadata reporting

The level of metadata reported by a geocoding system represents a critical factor that discriminates one geocoding system from another. As described above and in numerous research reports [29,45,46,55,56], geocoding quality indicators both at the per-record level (match type, match score, spatial accuracy) and overall process level (match rate) are important factors that describe how well a geocoding system performs. However, these are not the only forms of metadata that a geocoding system could report. Other metadata items that data producers and consumers could be concerned with include: (a) ***spatial confidence values*** – descriptions of the region size (geographic area) that a geocode output is known to fall within; (b) ***input address/matched address concordance*** – descriptions of which attributes of the input address were incorrect, incomplete, partially matched with corrections, not used in the matching process, etc. (Table 7).

**Table 7:** Geocoding system metadata metrics

**Metadata metric**	**Description**
**Spatial confidence values**	Does the system output spatial confidence intervals with each geocoded location? Example – Returning a buffer around the location within which the true geocode is known to be located
**Input address/Matched address concordance**	Does the system return an indication of the similarity between the input address requested and the address of the geographic reference feature matched? Example – Providing a list of the input address attributes that matched or did not match the address attributes associated geographic reference feature used for interpolation

### Capabilities

The baseline capabilities that a geocoding system provides are of paramount importance when evaluating the appropriateness of a geocoding system within a particular usage scenario. In addition to simply providing the ability to geocode a data set of input addresses, other capabilities that a geocoding system either does or does not provide include: (a) ***automatic batch geocoding*** – the ability to process a data file of records using a single process; (b) ***interactive review*** – the ability to perform manual review for non-matched records to attempt to determine a correct output geocode; (c) ***alias tables*** – the ability to incorporate tables of named places, common synonyms for street address attributes (suffixes, additional street names, etc.); and (d) ***weighted centroids*** – the ability to bias the output location based on a known distribution of a characteristic of interest such as the distribution of population or specific subsets of a population in an area (postcode, locality, etc.) (Table 8).

**Table 8:** Geocoding system capability metrics

**Capability metric**	**Description**
**Automated batch geocoding**	Does the system provide the ability to process a database of address records in batch mode? Example – Running the geocoding system over a database of records in a text file.
**Interactive review**	Does the system provide an interface that allows a user to review address records that do not match on a case-by-case basis? Example – Providing a graphical user interface (GUI) that allows a user to review geocoded results, make corrections and re-geocode.
**Alias tables**	Does the system provide the ability to add address alias tables into the geocoding process? Example – Providing the user with a capability to include the coordinates of named places, such as nursing homes, caravan parks, or prisons.
**Weighted centroids**	Does the system allow for the use of weighting schemes to bias the placement of centroid-level output? Example – Including a population density layer that moves the output of a postcode-level geocode closer to the location within the postcode that has the highest level of population density.

### End-user needs and expectations

Although the producers of geocoded data often make use of these data in-house within research projects and policy-making initiatives, it is often the case that the ultimate end-user of geocoded data may be in another area of an organization or be within a completely separate organization. In each case, the ***user expectations and requirements*** will vary by the end-user in terms of data quality. For example, an end-user computing disease rates at the state level would have an entirely different expectation for the accuracy of census unit assignments than one who sought to quantify individual-level exposure metrics at a micro-scale environmental level such as indoor residential exposure to pesticide. Similarly, the ***user knowledge of the geocoding process*** and ***user capacity to handle different levels of detail*** (metadata) about the geocoding process will vary by end-user group. For example, a policy-maker or legislative analyst may be overwhelmed if provided with detail about the input postal address attributes that did and did not match in a geocoded result. In contrast, a spatial statistician may wish to know that a proximate postcode was used to produce an output geocode for an input address where the input postcode was incorrect but the locality name was correct. The evaluation of a geocoding system must take into account the ***end-user needs, wants, and abilities*** to determine which features of a geocoding system are absolutely critical given the usage scenarios that are anticipated in the end-user communities which an organization’s geocoded data serve.

Similarly, the ***frequency of geocoding requests*** that are expected of a geocoding provider from end-users is an important aspect to consider, as is the ***volume of records that must be processed*** in each instance. A time consuming geocoding process that results in highly accurate results may be an acceptable option if the staff that must perform the geocoding are asked to do so infrequently or the data files are small. In contrast, organizations that must continually process large amounts of data or do so as part of an automated process simply cannot spend a great deal of time on a per-record basis, and as such, may be willing to sacrifice some level of accuracy or metadata for processing speed. These issues relate to the means by which the geocoding process is integrated into the organizational workflow, and whether or not the geocoding is ***performed on a per-project basis*** (one at a time), or if the process is tightly integrated into the mission of the organization and is ***an integral part*** of the services which the organization provides.

### Operating performance

The operating performance of a geocoding system defines characteristics of the geocoding system that affect how fast records can be processed. In most modern computing environments in use today, per-record processing speed is of little concern as many commercially available geocoding systems can process on the order of millions of records per hour. However, if large volumes of data must be continually processed or re-processed, speed may be an issue that can be used to discriminate between geocoding systems. An extreme example would be the need for real-time geocoding in a disaster or health emergency scenario such as a disease outbreak. Here, geocoded data are needed immediately to help resolve or understand a phenomenon as it is unfolding on the ground to assist in the decision-making process, determine where resources are needed, and identify a course of action to pursue to save lives and property.

## Materials

### Geocoding systems evaluated

Five desktop geocoding systems were evaluated. The geocoding systems used in this analysis were chosen from among the members of the Cooperative Research Centre for Spatial Information (CRC-SI). All 43 industrial partners of the CRC-SI were solicited to participate in this project through an expression of interest (EOI) process which requested information on the geocoding platforms provided by each partner. A set of conditions had to be met, the main one being that the platform had to be a stand-alone desktop system. Of those that responded, five were able to provide evaluation licenses and reference data that could be installed and tested as part of the evaluation. Four of the five systems represent state-of-the-art and well known commercial geocoding system offerings from companies that provide geocoding solutions for Australia and elsewhere in the world. All systems remain anonymous in this paper as per non-disclosure agreements and are indicated simply by the names “Geocoder A” through “Geocoder E”; position in this list of five (A – E) was assigned randomly. Each geocoding system was tested using each applicable reference data source and input data combination.

### Reference data sources

The reference data sources utilized in these experiments include the most up-to-date and accurate reference data files available for both the state of Western Australia (WA) and the entire country of Australia. The state-level files used were the Property Street Address (PSA) data files distributed by the Western Australian Land Information Authority (Landgate) [57]. These files include digital parcel boundaries (polygons) and parcel centroids (points) for all addresses in WA. Also used was an extension to the PSA, called PSA + within this report, which included spatially referenced place names also known as “alias tables”. These files are updated continuously and are the official government land records of the state which include the current postal address associated with each property.

The national-level files used in this study were the Geocoded National Address File (G-NAF) maintained and distributed by the Public Sector Mapping Agency (PSMA) Australia Limited [58]. These files are the nation-wide authoritative address data sources for the entire country of Australia. These data are collected from local, state, and national-level government agencies (including Landgate for WA), cleaned, integrated, and prepared for dissemination by PSMA. These data include the digital parcel boundaries (polygons) and parcel centroids (points) for nearly all addresses in Australia along with an associated current postal address associated with each property.

### Input data sources

The input data used for this study were chosen to represent three tiers of data types. The three types of data include health service utilization data, administrative list data, and gold standard data. The quality of these data range from exceptionally clean data that have been manually corrected which all geocoding systems should be able to process correctly, to exceptionally dirty data that are known to contain high levels of challenging geocoding scenarios which should cause errors in all geocoding systems. These diverse sets of input data with varying quality were chosen in order to compare how each of the geocoding systems could handle differently input data qualities and tease out the differences in how the internal geocoder processing techniques added to or subtracted from the resulting geocode quality produced by each system. Data use agreements with the data stewards responsible for the collection, curation, and maintenance of the data sets (including the gold standard data) used in this evaluation preclude the naming of the data set or the government agencies that provided them.

### Gold standard data

The gold standard data used for this study represent an exceptionally clean data set (data set A, n = 2,203) - a data source with no errors which should be correctly processed by all geocoding systems; non-matches in this system would be considered false negatives. This data set contained address data drawn from a previous, larger study. Each of the records in this data set represented an address that was not capable of being successfully geocoded using an automated geocoding system. These records were manually reviewed and processed to improve their output quality by verifying and/or correcting postal address attributes and the true location of the geocoded point following a method similar to that presented in Goldberg et al. (2008) [39]. The records were ground truthed using a variety of methods including aerial imagery, online “street view” software, contact of the parties responsible for the address to confirm address attributes, and linkage with official government records and public domain data sources. The result of these painstaking efforts was the construction of an input data set of addresses with attribute data (number, street name, suffix, locality, postcode, etc.) that were manually confirmed to be correct.

### Administrative data

The administrative data set (data set B, n = 1,364,058) used for this study was drawn from official records of a large WA administrative database. These data contain the official addresses of a subset of residents of WA, and represent input address data that should be of fairly high quality. These data are representative of many administrative lists that are used to send out government mailings, confirm postal delivery addresses, and other essential government services.

### Health service utilization data

The health service utilization data set (data set C, n = 1,264,941) used for this study was chosen to represent a data source with numerous errors in the input address which would be the most difficult to geocode and result in the highest number of non-matches, false positive matches (incorrect matches), and false negative non-matches (incorrect non-matches). These data were drawn from the health service utilization records of a specific Western Australian health agency and are representative of the quality of data that occur when data are collected through a patient-facing organization where the patient self-reports his/her postal address.

The primary challenges of these data were threefold –

• Blank fields in addresses resulting in input data with limited input address fields, sometimes with just a locality and/or just a postcode;

• Named places such as prisons, nursing homes, and Aboriginal communities, instead of street addresses; and

• Historical data which includes many versions of data input systems all of which captured data in different ways ranging over a number of years.

Variations to data collection procedures through time include:

• Truncations to save characters;

• Transposition and introduction of new fields as user interfaces were updated; and

• Use of various codes for unknown/missing information (e.g., entering postcode 9999 when the postcode was unknown versus leaving it blank or entering 0000).

These data included numerous types of other frequently occurring errors including misspellings to all components of the input address (number, street name, suffix, locality, postcode, etc.), the use of incorrect locality names and postcodes, and all combinations of missing attributes for all fields of the input address.

### Experimental design

The experiments performed for this research attempted to apply the framework and metrics described above in the context of the Western Australia (WA) Department of Health (DoH) as a test-case for evaluating their applicability for comparing a set of available geocoding platforms. To do so, the characteristics of each geocoding system were assessed across each aspect of the evaluation framework presented earlier. Table 9 was constructed in consultation with the WA DoH as the features and capabilities of geocoding systems which were important to the organization. Each system was evaluated based on published literature and documentation of the geocoding systems. Additional communication with each vendor was necessary to determine all capabilities because not all vendors use the same terminology for all items.

**Table 9:** Operational capabilities results

**Evaluation metric**	**Geocoder A**	**Geocoder B**	**Geocoder C**	**Geocoder D**	**Geocoder E**
**User-defined reference data layers license fee (Y/N)**	Yes	Yes	Yes	Yes	No
**Specialized address parsing rules (Y/N)**	Yes	Yes	Yes	Yes	No
**Specialized matching algorithms (Y/N)**	Yes	No	No	Yes	Yes
**Specialized feature selection hierarchies (Y/N)**	Yes	Yes*	Yes*	Yes	Yes
**Integration**					
**Operating system support (Y/N)**	Yes (Unix)	No (Windows)	No (Windows)	Yes (Windows, Unix, Linux)	Windows
**Native system and workflow integration (Y/N)**	Yes	No	No	No	No
**Batch mode (Y/N)**	Yes	Yes	Yes	Yes	Yes
**Interactive mode (Y/N)**	Yes	No	No	Yes	No
**Manual review (Y/N)**	Yes	Yes	Yes	Yes	No
**Confidentiality maintained (Y/N)**	Yes	Yes	Yes	Yes	Yes
**Desktop version(Y/N)**	Yes	Yes	Yes	Yes	Yes
**In-house server version (Y/N)**	Yes	No	No	Yes	No
**Online API version (Y/N)**	No	Yes	Yes	Yes	No
**Metadata**					
**Match rate (Y/N)**	Yes	Yes	Yes	Yes	Yes
**Match type (Y/N)**	No	No	No	Yes	Yes
**Match score (Y/N)**	Yes	Yes	Yes	Available (not by default)	No
**Spatial confidence (Y/N)**	Yes	No	No	Yes	No
**Input address/matched address concordance (Y/N)**	Yes	Yes	Yes	Yes	Yes
**Capabilities**					
**Automatic batch geocoding (Y/N)**	Yes	Yes	Yes	Yes	Yes
**Manual review (Y/N)**	Yes	Yes	Yes	Yes	Yes
**Alias tables (Y/N)**	Yes	Yes	Yes	Yes	No
**Weighted centroids (Y/N)**	No	No	No	Yes	No
**Nearby address matching (Y/N)**	Yes	No	No	Yes	Yes

* Only if street centroids, suburb and postcode reference data are available.

The project team attempted to install each system 'out-of-the-box’ without customization as much as possible. This included importing reference data layers into some of the systems as necessary, i.e., those that did not include the reference data as part of the software, instead requiring a geocoding reference data layer to be constructed or specified. An exception to this is the programming required to install Geocoder A which is described below.

The three input data sets were batch-processed through each of the geocoding systems on the same team-member’s computer in sequence. No data filtering, data cleansing, address standardization, or address normalization operations were applied to any of the input data prior to geocoding being performed. All data were processed directly as received from the data custodians although the first step in most batch geocoding systems is to standardize and normalize the input data internally within the geocoding system [59].

The experiments performed controlled for differences in geocoding quality due to the three main components of geocoding systems: (a) input data quality; (b) geocoding algorithms which include all components of the geocoding system that are beyond the control of a geocode user – address standardization and normalization, feature matching, and feature interpolation; and (c) the reference data layers used. To do so, each of these three components was evaluated separately by constructing usage scenarios that attempted to vary one aspect and keep the other two constant. Each of these axes was tested by varying one and holding the other two constant.

For example, to test the effect of input data quality across each geocoding system, all three data sets where processed by each geocoder using the same reference data sources (as could be achieved based on different reference data set support per geocoder). Holding the reference data sets static and changing the input data set allowed for analysis of the overall effect of excellent (Gold Standard), moderate (Administrative), and poor (Health) quality data on each geocoding system. Similarly, the effect of reference data set usage was evaluated by holding the input data set constant and processing it with different combinations of reference data layers, per geocoding system.

## Results and discussion

### Reference data layers

Table 10 lists the supported reference data layers per each geocoding system. Geocoding systems were evaluated on their ability to support the G-NAF, PSA, and PSA + data (PSA with additional alias names contained). Only one of the geocoding systems tested, Geocoder B, could support all three reference data layers without any additional development work and associated costs. All four other geocoding systems *could* have supported the additional reference data layers, but this would have required specialized customization and/or development work by the system providers which was beyond the budget and scope of the current research.

**Table 10:** Reference data set support and setup time

**Geocoder**	**G-NAF**	**PSA**	**PSA+**
**A**	No	Yes – 20 mins	Yes – 3 weeks
**B**	Yes – 1 day	Yes – < 5 mins	Yes– < 5 mins
**C**	No	Yes – < 5 mins	Yes – < 5 mins
**D**	Yes – < 5 mins	Yes – < 5 mins	No
**E**	Yes – < 5 mins	No	No

The most striking result shown in Table 10 is that fact that Geocoder A took several weeks to import the latest data layers available. This amount of time was needed for specialized staff to perform custom programming to build in support for modern data formats (shapefiles and geodatabases instead of older formats). This update represented a large one-time investment for the Geocoder A system.

### Processing time

Table 11 lists processing times required to geocode all records within each input data set using each applicable reference data layer within each geocoding system. In general, all but Geocoder A processed data at roughly the same speed given the number of records. In all instances, the processing speed was deemed acceptable for the number of records due to the fact that they were processed in batch for non-real time purposes.

**Table 11:** Processing time by geocoding system, reference data set, and input data set

**Dataset**	**Reference data**	**Geocoder A**	**Geocoder B**	**Geocoder C**	**Geocoder D**	**Geocoder E**
**A – Gold Standard**	PSA	<2 m	<2 m	<2 m	<2 m	-
**(n = 2,203)**	PSA+	<2 m	<2 m	<2 m	-	-
	G-NAF	-	<2 m	-	<2 m	<2 m
**B – Administrative**	PSA	45 m	19 m	13 m	17 m	-
**(n = 1,364,058)**	PSA+	39 m	19 m	12 m	-	-
	G-NAF	-	24 m	-	34 m	13 m
**C – Health**	PSA	55 m	16 m	16 m	25 m	-
**(n = 998,066 )**	PSA+	2 h 25 m	22 m	17 m	-	-
	G-NAF	-	19 m	-	30 m	23 m

### Operating metric comparison

Each of the five geocoding systems was evaluated using the operational capabilities described above. Table 9 displays the evaluation results of each geocoding system against an operational capabilities matrix derived from the above metrics which were deemed important within the context of the WA DoH usage scenario. Using this data, it is possible to make comparative assessments of the match rates across the varying geocoding systems. It is expected that different organizations and/or usage scenarios would choose or develop alternative/additional framework metrics to evaluate geocoding systems based on the most important operational and performance needs of the organization.

### Match type and match rate summary

The match type and match rate results from each of the five geocoding systems are displayed in Tables 12, 13, and 14. These results are divided between input data set and applicable reference data layers for each geocoding system. The results are divided into 'Parcel’ level match and 'Non-Parcel’ level match. For the geocoding systems that indicate a match type (Geocoder D and Geocoder E), this output was used directly to determine 'Parcel’ level and 'Non-Parcel’ level matches. For those systems which did not indicate a type of match, but instead assign a match score only – a level of similarity between the input and reference data – (Geocoder A, B, and C) thresholds of match scores were selected to represent 'Parcel’ level and 'Non-Parcel’ level geocodes.

**Table 12:** Input data A (Gold standard) match type and match rate summary (n = 2203 records)

**Geocoder**	**Reference**	**'Parcel’ level**		**'Non-parcel’ level**		**Geocoded**	
	**Data**	**N**	**%**	**N**	**%**	**N**	**%**
**A**	**G-NAF**	-	-	-	-	-	-
	**PSA**	1875	85.1	303	13.8	2178	98.9
	**PSA+**	1875	85.1	303	13.8	2178	98.9
**B**	**G-NAF**	1765	80.1	67	3.0	1832	83.2
	**PSA**	1624	73.7	77	3.5	1701	77.2
	**PSA+**	1624	73.7	77	3.5	1701	77.2
**C**	**G-NAF**	-	-	-	-	-	-
	**PSA**	1696	77.0	21	1.0	1717	77.9
	**PSA+**	1696	77.0	21	1.0	1717	77.9
**D**	**G-NAF**	1959	88.9	236	10.7	2195	99.6
	**PSA**	1938	88.0	257	11.7	2195	99.6
	**PSA+**	-	-	-	-	-	-
**E**	**G-NAF**	1991	90.4	212	9.6	2203	100.0
	**PSA**	-	-	-	-	-	-
	**PSA+**	-	-	-	-	-	-

**Table 13:** Input data B (Administrative) match type and match rate summary (n = 1364058 records)

**Geocoder**	**Reference**	**'Parcel’ level**		**'Non-parcel’ level**		**Geocoded**	
	**Data**	**N**	**%**	**N**	**%**	**N**	**%**
**A**	**G-NAF**	-	-	-	-	-	-
	**PSA**	1306310	95.8	55907	4.1	1362217	99.9
	**PSA+**	1313046	96.3	49805	3.7	1362851	99.9
**B**	**G-NAF**	1136220	83.3	36915	2.7	1173135	86.0
	**PSA**	1165034	85.4	58664	4.3	1223698	89.7
	**PSA+**	1165034	85.4	58664	4.3	1223698	89.7
**C**	**G-NAF**	-	-	-	-	-	-
	**PSA**	1219245	89.4	21932	1.6	1241177	91.0
	**PSA+**	1219245	89.4	21932	1.6	1241177	91.0
**D**	**G-NAF**	1318281	96.6	43825	3.2	1362106	99.9
	**PSA**	1325911	97.2	35442	2.6	1361353	99.8
	**PSA+**	-	-	-	-	-	-
**E**	**G-NAF**	1329627	97.5	34431	2.5	1364058	100.0
	**PSA**	-	-	-	-	-	-
	**PSA+**	-	-	-	-	-	-

**Table 14:** Input data C (Health) match type and match rate summary (n = 998066 records)

**Geocoder**	**Reference**	**'Parcel’ level**		**'Non-parcel’ level**		**Geocoded**	
	**Data**	**N**	**%**	**N**	**%**	**N**	**%**
**A**	**G-NAF**	-	-	-	-	-	-
	**PSA**	712645	71.4	149309	15.0	861954	86.4
	**PSA+**	724326	72.6	145595	14.6	869921	87.2
**B**	**G-NAF**	446182	44.7	101049	10.1	547231	54.8
	**PSA**	486188	48.7	78508	7.9	564696	56.6
	**PSA+**	486188	48.7	78508	7.9	564696	56.6
**C**	**G-NAF**	-	-	-	-	-	-
	**PSA**	440062	44.1	27806	2.8	467868	46.9
	**PSA+**	440062	44.1	27806	2.8	467868	46.9
**D**	**G-NAF**	734518	73.6	211175	21.2	945693	94.8
	**PSA**	725115	72.7	217965	21.8	943080	94.5
	**PSA+**	-	-	-	-	-	-
**E**	**G-NAF**	716241	71.8	271326	27.2	987567	98.9
	**PSA**	-	-	-	-	-	-
	**PSA+**	-	-	-	-	-	-

### Interpretation and discussion

Functionally, the biggest issues which affected geocoder performance were 1) the ability to include additional reference data layers; and 2) the ability to include alias tables. The geocoding systems evaluated in this project spanned the spectrum of flexibility in this regard. For example, Geocoder A included alias tables but could not include the G-NAF data. The Geocoding B and C systems allowed users to include any parcel based file (G-NAF and PSA) but encountered challenges including alias tables, although the documentation reports that these could be added if data layers can be obtained and formatted properly. Geocoder D could include both G-NAF and PSA but could not utilize alias tables (PSA+), while Geocoder E could only utilize G-NAF without costly development at the Geocoder E organization to include the PSA or PSA + files.

The impact of including alias tables was evident when inspecting the results of the data set C (Health). Geocoder A was the only one that could include these and, as a result, was the only system that performed well on this data set. These data were known to include a high degree of named places such as nursing homes and caravan parks which are not geocodable without the inclusion of alias tables. Conversely, the lack of support for G-NAF data did not appear to be a major problem that affected the quality of Geocode A performance. Australia has a unique addressing system, which is why address-range geocoding systems [32] are used less frequently than parcel or address-point based systems [28]. The increase in quality of output data from systems which included alias tables may also be an artefact of the addressing system used in Australia.

Other differences between geocoding systems related to the amount and quality of metadata returned along with a result. Geocoder A returned a quantitative value describing an area within which the geocode is considered to fall. Geocoders B and C, on the other hand, return a match score describing the similarity between the input address and the geographic feature that was matched to. Both Geocoders D and E provided a greater degree of detail about the specific attributes of the input address that matched the reference feature, as well as details about the geographic level of the match and/or mismatch of these attributes. These types of details permit a user a greater understanding of the match quality than a simple match score, but do not provide a quantitative spatial measure with which to understand how spatially in/accurate an output geocode could be.

As noted above, the most pronounced operational distinction between geocoding systems was the setup time necessary to build a geocoding system and the amount of specialized skill required to maintain the system. Geocoder A was the most difficult to setup for the evaluation due to required programming. With in-depth documentation and the upgrade to modern data formats completed, this time may be reduced going forward, but in all cases it will remain a time-consuming task which requires specially-trained staff to be in-house experts. All other geocoding systems could be installed, setup, and run in a fraction of the time required to update Geocoder A to the latest version of the PSA and PSA + data.

As results demonstrate, the overall quality of input data had a pronounced impact on the quality of the output results. Data set C (Health), known to include a high degree of difficult cases such as named places, resulted in the worst output geocode quality across systems. Similarly, the high quality data (Gold Standard) resulted in the highest quality matches. These results are indicative of the fact that input data quality matters. The results demonstrate that, wherever possible, input data should be cleaned as close to the source of collection as possible.

To account for these and other errors in the input data, the geocoding algorithms, or the reference data used by the geocoding systems, manual geocoding may need to be performed to correct or otherwise assign records that could not be processed. The degree to which manual geocoding procedures are linked into the automated geocode process varied between the geocoding systems. Geocoders A, B and C included a post-processing step to automatically update the output files. These geocoding systems offer the ability for a user to review specific types of records, make corrections, and offer candidate matches. Geocoders A, B and C take roughly the same amount of time to process individual records and offer the key benefit that they work directly on the output data file and update an output geocode’s value once it is reprocessed so that table joining between processed and post-processed data are not required.

A central question a reader should be asking at this point is: How should the findings presented here, or a similar evaluation performed by another organization or on a different set of geocoders, be used to decide which geocoding system should be the correct choice? The answer is unfortunately not straightforward. As discussed above, every organization is different and will value certain aspects or capabilities of geocoding systems more or less than another organization. Every organization will have different strengths (in-house programming skills, for example) or resources (access to reference data layers, for example) which will affect the cost-benefit equation used to rank geocoding choices.

One potential and simple method that could be used to determine the correct choice would be to borrow from suitability research [60]. First determine which geocoding system criteria are important and which are not. This list may include each of the criteria we have described here, a subset thereof, or others that may be important to an organization but were not listed in the set presented here. Next, assign a relative weight of importance to each of these criteria so that some things are more important than others – i.e., nice-to-have’s versus must-have’s. Next perform a capability analysis across each of the criteria for each geocoder and assign the appropriate binary (1/0) or scaled scores depending on the data type determined or each criteria (i.e., nominal, ordinal, ratio, or interval data). These analyses could simply assess capabilities like those listed in Table 2, 3, 4, 5, 6, 7 and 8 or they could include large-scale geocoding system performance tests as we have done here in order to determine a subset of the performance metrics listed in Table 1.

Once all geocoders are scored across all criteria, the most promising option should rise to the top. A central purpose of performing the current research to develop a methodology of assessing geocoding systems was to enable just this type of analysis for making geocoding system decision at the WA DoH. However, the exact criteria and their weightings to be used in the WA DoH decision-making process are not presented here; instead just the methodology organizations could follow to do similar tasks on their own.

### Evaluation framework limitations

Not all enumerations of all geocoding test scenarios could be performed due to limitations in the flexibility of various geocoding systems. For example, the use of alias tables could not be turned off in Geocoder A; nor could G-NAF data be loaded. This mean that results from Geocoder A could not be included in the analyses that determined the benefits of (a) local versus national reference data files, and (b) the use of alias tables versus the non-use. Similarly, all but Geocoder B had limitations to the types of reference data layers that could be utilized.

## Conclusion

The central goal of this paper was to present an objective methodology for comparing geocoding systems. The purpose of such a methodology is to assist in the decision-making process when evaluating the performance and utility of a range of geocoding systems. The particular evaluation context investigated here was a case study involving a typical geocoding use-case performed within a large government agency for which geocoding is a mission-critical task. This organizational case study and the current techniques employed within the organization geocoding can, in many ways, be seen as representative of many large organizations within the public or private sector around the globe. Like others, the organization in this study has spent a considerable amount of time and effort developing a geocoding process that is integral to its core business. The geocoding system currently in place is tightly integrated into other core operational and workflow systems, has been highly tailored to the type of data it encounters, and has produced results of sufficient quality for a range of users.

Notwithstanding current arrangements, there are many reasons why decision-makers may wish to perform an analysis of other available geocoding platforms, in part to identify other alternatives that might work better, be cheaper, or offer an enhanced set of services. In particular, government systems continue to be enhanced, the cost of hardware continues to drop, and data processing operations within government agencies are continually reviewed for opportunities for modernization and streamlining to better serve the public at lower costs. Government departments and private industry continually re-evaluate practices to seek better ways of operating.

The purpose of the methodology developed here is to act as a tool for gathering data for use by decision-makers. The quantitative data generated by the framework presented here must be used in coordination with other strategic initiatives within an organization in order to make the most informed and rational decision, given the specific context and plan of an organization.

## Endnotes

^a^http://geocoder.us/

^b^http://www.census.gov/geo/www/tiger/

^c^http://www.pagcgeo.org/

## Competing interests

The authors declare they have no competing interests.

## Authors’ contributions

DWG conceived of the study design, performed data analysis, and drafted the manuscript. MB contributed to study design, ran the experiments, and performed data analysis. NM contributed to study design and facilitated the acquisition of test software and reference data. GC and DR contributed to study design, facilitated the acquisition and preparation of study data, enabled study execution. AF, JB & JS contributed to study design, data preparation, and analysis. All authors contributed to and reviewed the final manuscript.
